# Dosimetric evaluation of a novel automated noncoplanar volumetric modulated arc therapy technique for treating optic nerve sheath meningiomas

**DOI:** 10.3389/fonc.2025.1531918

**Published:** 2025-06-27

**Authors:** Zhenyu Xiong, Chingyun Cheng, Lili Zhou, Brett Eckroate, Loren Bell, Fredrick Warburton, David Huang, Sabin B. Motwani, Charles S. Cathcart, Ke Nie, Ning Yue, Yin Zhang

**Affiliations:** ^1^ Department of Radiation Oncology, Rutgers Cancer Institute of New Jersey, Rutgers, The State University of New Jersey, New Brunswick, NJ, United States; ^2^ Robert Wood Johnson Medical School, Rutgers, The State University of New Jersey, New Brunswick, NJ, United States; ^3^ Department of Radiation Oncology, RWJBarnabas Health, West Orange, NJ, United States; ^4^ Department of Human Oncology, School of Medicine and Public Health, University of Wisconsin-Madison, Madison, WI, United States

**Keywords:** optic nerve sheath meningioma, HyperArc, volumetric modulated arc therapy, dosimetric comparison, radiotherapy

## Abstract

**Purpose:**

This study aimed to evaluate the dosimetric outcomes for the target and organs at risk (OARs) in patients with optic nerve sheath meningiomas (ONSMs), comparing HyperArc (HA), a novel automated noncoplanar volumetric modulated arc therapy (VMAT) technique, with two other advanced VMAT techniques.

**Methods:**

Nine patients with ONSMs were re-planned using three radiotherapy techniques: HA employing four preconfigured noncoplanar partial arcs on the Varian TrueBeam, a two-arc coplanar VMAT on the Varian TrueBeam (TB-VMAT), and a two-arc coplanar VMAT on the Varian Halcyon (HAL-VMAT). All treatment plans aimed to deliver 50.4 Gy in 28 fractions to the planning target volume (PTV) while minimizing dose to OARs. The planning process began by applying identical preset optimization templates for each plan, followed by iterative refinements of objectives and priorities to accommodate individual plan requirements. All plans were normalized to ensure that 100% of the prescription dose covered 95% of the PTV. Dosimetric evaluation included PTV metrics (D_98%_, D_mean_, D_max_, and D_min_), the Paddick Conformity Index (PCI), the International Commission on Radiation Units and Measurements Report 83 (ICRU-83) homogeneity index (HI), the gradient index (GI), and doses to OARs for each technique. Statistical significance was assessed using the Wilcoxon signed-rank test with a p-value threshold of < 0.05.

**Results:**

HA plans demonstrated superior dosimetric indices for PTV, as indicated by the highest D_98%_ (50.24 ± 0.05 Gy) and the lowest D_max_ (53.20 ± 0.23 Gy), HI (0.04 ± 0.00), and GI (3.56 ± 0.58) values (p < 0.05). These results indicated superior target coverage and a more homogeneous dose distribution. Furthermore, HA plans achieved the lowest maximum dose values for the following OARs: lenses, hippocampi, contralateral optic nerve, and contralateral retina (p < 0.05), thereby optimally sparing these critical structures. No significant differences were observed across techniques regarding D_mean_, D_min_, PCI, or maximum dose to the ipsilateral optic nerve, ipsilateral retina, and optic chiasm.

**Conclusions:**

HA plans demonstrated superior dosimetric performance, ensuring adequate target coverage, reduced PTV hotspots, and better OAR protection compared to coplanar VMAT plans on the Varian TrueBeam and Halcyon. These advantages suggest that

## Introduction

Optic nerve sheath meningiomas (ONSMs) are rare benign tumors originating from arachnoid meningoendothelial cells and affect the anterior visual pathway (1). They constitute up to 2% of all meningiomas and are the second most common primary optic nerve tumors (2). Clinical manifestations at diagnosis predominantly include compromised visual acuity—ranging from partial impairment to complete vision loss—as well as visual field deficits, color perception abnormalities, and exophthalmos. Management of ONSMs remains particularly challenging due to their intimate relationship with the optic nerve. Surgical interventions are generally limited to cases exhibiting severe visual deterioration or pronounced exophthalmos, as the characteristic circumferential growth of meningiomas around the nerve often precludes total resection without risking substantial damage to the optic nerve or adjacent vasculature (3).

Radiotherapy has increasingly become the treatment of choice for ONSMs due to its favorable outcomes in preserving or improving visual acuity and visual fields (4–6). Currently, several radiation modalities are employed to treat ONSMs, including two-dimensional radiation therapy (2DRT) (7), three-dimensional conformal radiation therapy (3DCRT) (8), intensity-modulated radiation therapy (IMRT) (9), fractionated stereotactic radiation therapy (FSRT) (10), and proton beam therapy (PBT) (11). ONSMs involve or are adjacent to various critical organs at risk (OARs), such as the optic nerves, optic chiasm, lenses, and retinas. Advances in radiotherapy technology have led to newer modalities like FSRT and IMRT, which provide better radiation dose conformity leading to fewer complications compared to conventional 3DCRT (9, 12). For ONSMs, one of the main objectives in patient management is to preserve or improve visual function, as these benign tumors do not threaten patient survival and exhibit slow growth with high local control rates after treatment (13). Therefore, in radiotherapy treatment planning for ONSMs, radiation dose to OARs must be strictly prioritized over dose coverage of the target volume.

HyperArc (HA) (Varian Medical Systems, Palo Alto, CA), a relatively new noncoplanar VMAT technique with automated delivery, has been developed to generate steep dose gradients with efficient planning workflow compared to conventional VMAT methods, particularly for stereotactic radiosurgery (SRS) or stereotactic radiation therapy (SRT) treatment. HA utilizes both coplanar and noncoplanar arcs to treat single or multiple intracranial targets with a single isocenter. It aims to improve planning workflow by automatically placing the isocenter within the patient protection zone and optimizing collimator angles of the preconfigured noncoplanar arcs based on the location and geometry of the PTV(s). HA also provides safe and efficient workflow for treatment delivery by offering a virtual dry run function in the treatment planning system (TPS) to reduce the risk of collisions and automating VMAT arcs delivery.

HA has proven effective as an SRS/SRT technique, providing high conformity of target dose and low doses to OARs in patients with single or multiple brain metastases (14–17). Recently, HA has also been applied to other tumors, such as glioblastoma multiforme (GBM) (18) and head and neck cancers (19, 20), due to its superior dose distribution. These studies showed that HA can achieve better target coverage and OAR sparing. However, no study has investigated whether doses to OARs could be reduced while maintaining target coverage when using HA technique in treating patients with ONSMs.

The aim of the present study was to evaluate the dosimetric outcomes for the target and OARs using HA in comparison with two others advanced VMAT techniques—a two-arc coplanar VMAT on the Varian TrueBeam (TB-VMAT) and a two-arc coplanar VMAT on the Varian Halcyon (HAL-VMAT)—in patients with ONSMs.

## Materials and methods

### Patient selection and simulation

A cohort of nine patients diagnosed with ONSMs at our institution were enrolled in this retrospective study, which was approved by our institutional review board (IRB). All patients were previously treated with coplanar VMAT on the Varian TrueBeam or Halcyon systems between 2020 and 2024. Five patients had lesions on the right side (55.6%), and the other four had lesions on the left side (44.4%). The involvement of the optic canal was both canalicular and orbital in more than half of the cases (55.6%). The demographics and characteristics of these patients are listed in **Table 1**.

**Table 1:** Summary of clinical characteristics for 9 patients with ONSM.

Characteristics	
Patient number	9
Gender	
Male	2
Female	7
Age (y)	
Median	54
Range	23-64
Side	
Left	4
Right	5
Involvement of optic canal	
Canalicular	2
Orbital	2
Both	5
GTV (cm^3^)	
Median	1.7
Range	0.3-4.3
PTV (cm^3^)	
Median	8.1
Range	2.9-28.0

Computed tomography (CT) simulations (Brilliance Big Bore, Philips Healthcare) were performed with 1.5 mm slice thickness and a resolution of 512 × 512 pixels. Magnetic resonance imaging (MRI) scans were also obtained. Rigid image registration was performed to align MRI scans with the corresponding planning CT images in the TPS (Eclipse 16.1; Varian Medical Systems). The gross tumor volume (GTV) was delineated based on all available clinical and imaging information. The planning target volumes (PTVs) were created by symmetrically extending the GTVs by 5–7 mm. The mean PTV size was 8.1 ± 10.6 cm³ (range: 2.9–28 cm³). The target volumes and OARs, such as the lenses, optic nerves, optic chiasm, retinas, and hippocampi, were contoured by three experienced radiation oncologists at our institution. The Exact IGRT Couch (Thin) was inserted for the TB-VMAT plans, and the Halcyon Couch was inserted for the HAL-VMAT plans.

### Treatment planning

**HA:** Unlike conventional coplanar VMAT, HA uses a fixed geometry setup with four preconfigured arcs: one full or half coplanar arc with couch rotation of 0° and three half noncoplanar arcs with couch rotations of 45°, 315° and 90°or 270°. In this study, four half arcs with couch rotations at 0°, 45°, 90° (for left ONSMs) or 270° (for right ONSMs), and 315° were used. The treatment planning process, including the positioning of the isocenter and selection of collimator angles, was automatically designed according to the position and shape of the targets. To reduce the risk of collision due to the automated delivery nature of HA, the permissible isocenter locations are limited to within a specific patient protection zone.

**Figure 1:**
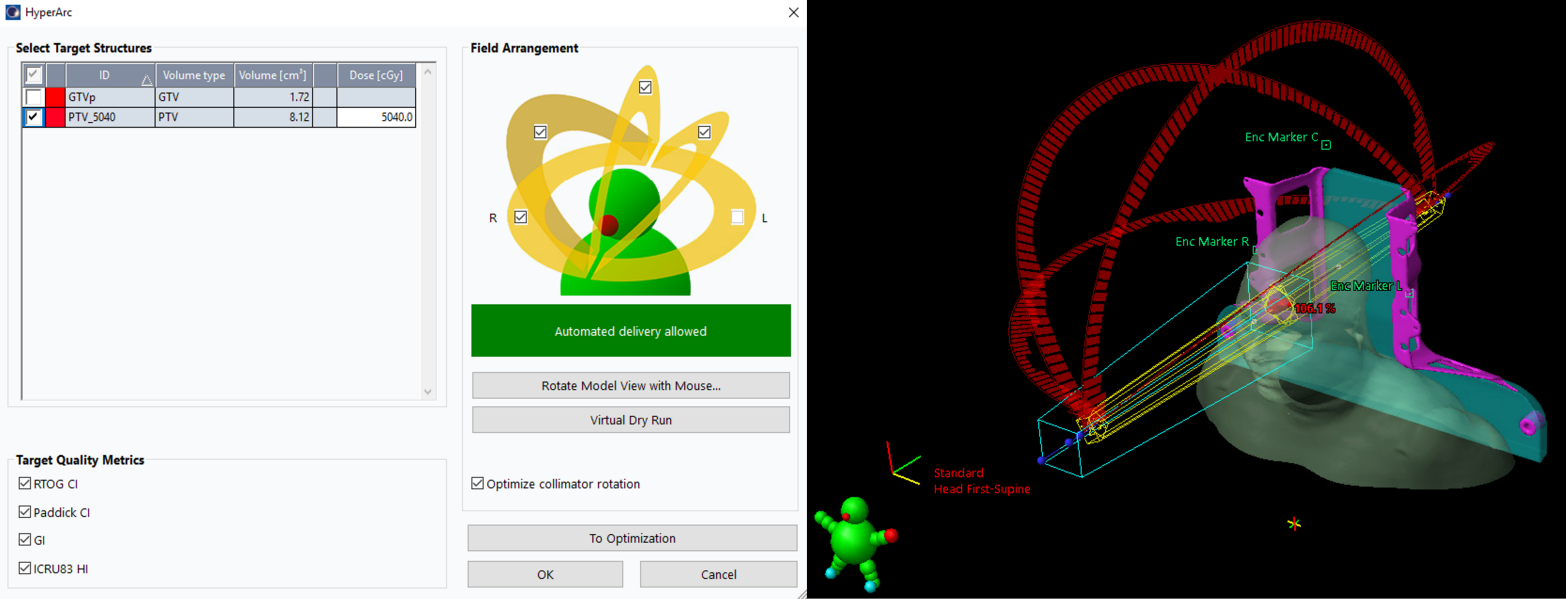
Users’ interface to select target(s), isocenter, arcs, and collimator angles for the HA plans in Eclipse. With the virtual dry run feature, HA can perform a collision check to the selected arcs before the optimization.

In our study, the structures of the Encompass (QFix, Avondale, PA) SRS immobilization system were inserted into the CT images only for the HA plans. All four half arcs were used with an automatically selected isocenter, optimized collimator rotation, and jaw tracking, as demonstrated in **Figure 1**. A virtual dry run was performed in the TPS to predict clearance between the patient and the linear accelerator (Linac) using the planning CT dataset. The SRS normal tissue objective (SRS NTO) was not used for optimization in this study since radiotherapy for ONSMs is not an SRS treatment approach. The optic nerve, optic chiasm, and retina often overlapped with the PTV. Therefore, using the SRS NTO could result in higher doses within the PTV (higher HI), leading to increased doses to these overlapping OARs. For the HA plans, 6 MV flattening filter (FF) photon beam with a dose rate of 600 MU/min from a Varian TrueBeam Linac equipped with a 120-leaf Millennium multileaf collimator (MLC) was used.

**TB-VMAT:** For the TB-VMAT plans, 6 MV FF photon beam was used with a dose rate of 600 MU/min from a Varian TrueBeam Linac equipped with a 120-leaf Millennium MLC. Two full arcs were employed with collimator angles of 30° and 330°.

**HAL-VMAT:** For the HAL-VMAT plans, 6 MV flattening filter-free (FFF) photon beam with a dose rate of 800 MU/min, which is the only available photon beam energy on the machine, was used from a Varian Halcyon Linac equipped with a 114-leaf SX2 MLC. Two full arcs were employed with collimator angles of 30° and 330°.

The prescription dose was 50.4 Gy in 28 fractions and the plans were normalized so that 100% of the prescription dose covered 95% of the PTV, ensuring fair and meaningful comparisons of coverage parameters such as PTV D_98%_ and PTV D_min_. The Anisotropic Analytical Algorithm (AAA, version 16.1) with a 2.5 mm calculation grid size was utilized for dose calculations in Eclipse to reflect current clinical practice at our institution and ensure consistency with previously delivered clinical plans for direct dosimetric comparisons. Each plan initially employed standardized preset optimization templates with identical objectives and priorities (**Table 2**) to ensure consistency and enable fair comparisons. Subsequently, we iteratively refined these objectives and priorities to address the specific requirements of each individual plan. The jaw tracking function was enabled in the optimization process for the HA and TB-VMAT plans.

**Table 2:** Optimization objectives and priorities used for each patient for all three techniques for target and normal structures.

Structure	Objective	Priority
PTV	D_max_ < 53 Gy	100
	D_100%_ > 50.4 Gy	100
Optic Nerve	D_max_ < 50.4 Gy	50
Optic Chiasm	D_max_ < 50.4 Gy	50
Retina	D_max_ < 50.4 Gy	50
Brain	D_max_ < 50.4 Gy	30
Lens	D_max_ < 8 Gy	30
NTO	Automatic	75

### Dosimetric parameters and plan quality analysis

Plan evaluation was performed using dosimetric parameters calculated from the dose-volume histogram (DVH) for the PTV and the following OARs: lenses, optic nerves, optic chiasm, retinas, and hippocampi. Target coverage was assessed by the minimum dose (PTV D_min_) and the minimum dose covering 98% of the PTV volume (PTV D_98%_). The maximum dose for the PTV (PTV D_max_) was also evaluated, along with the mean dose of the PTV (PTV D_mean_). The maximum doses to the contralateral and ipsilateral lenses, optic nerves, retinas, and hippocampi, as well as to the optic chiasm, were also evaluated.

The Paddick Conformity Index (PCI) (21) was calculated for each plan as follows:


$$\text{PCI}=\frac{{\left(\text{TV}\cap \text{PIV}\right)}^{2}}{\text{TV}\times \text{PIV}}$$

where TV is the target volume (PTV), PIV is the volume that actually receives the prescribed dose of radiation, and TV ∩ PIV is the target volume covered by the prescription isodose. The PCI defines how precisely the prescription dose distribution matches the target, with an ideal value of 1.

The ICRU-83 Homogeneity Index (HI) (22) was calculated using:


$$\text{HI}=\frac{{\text{D}}_{2\%}-{\text{D}}_{98\%}}{{\text{D}}_{50\%}}$$

where D_2%_ is the highest dose received by 2% of the PTV volume, D_98%_ is the highest dose received by 98% of the PTV volume, and D_50%_ is the highest dose received by 50% of the PTV volume. A smaller HI value indicates a more homogeneous dose distribution, with an ideal value of 0.

The gradient index (GI) (23) was calculated as:


$$\text{GI}=\frac{\text{PIV}50\%}{\text{PIV}100\%}$$

The GI characterizes the slope of the dose gradient between the prescribed dose level and 50% of this dose, describing the steepness of the dose gradient from the PIV to the surrounding tissue. The goal is to achieve the lowest possible GI value.

In addition to the evaluation of the dosimetric parameters mentioned above, the total number of monitor units (MUs), beam-on time (BOT), and delivery quality assurance (QA) results were also assessed. The BOTs were recorded during the delivery of the QA. The delivery QA was measured using portal dosimetry. The quantitative evaluation of dosimetric accuracy was performed using gamma analysis criteria: 3% dose difference, 2 mm distance-to-agreement, and a low dose threshold at 10% of maximum signal.

### Statistical analysis

**Figure 2:**
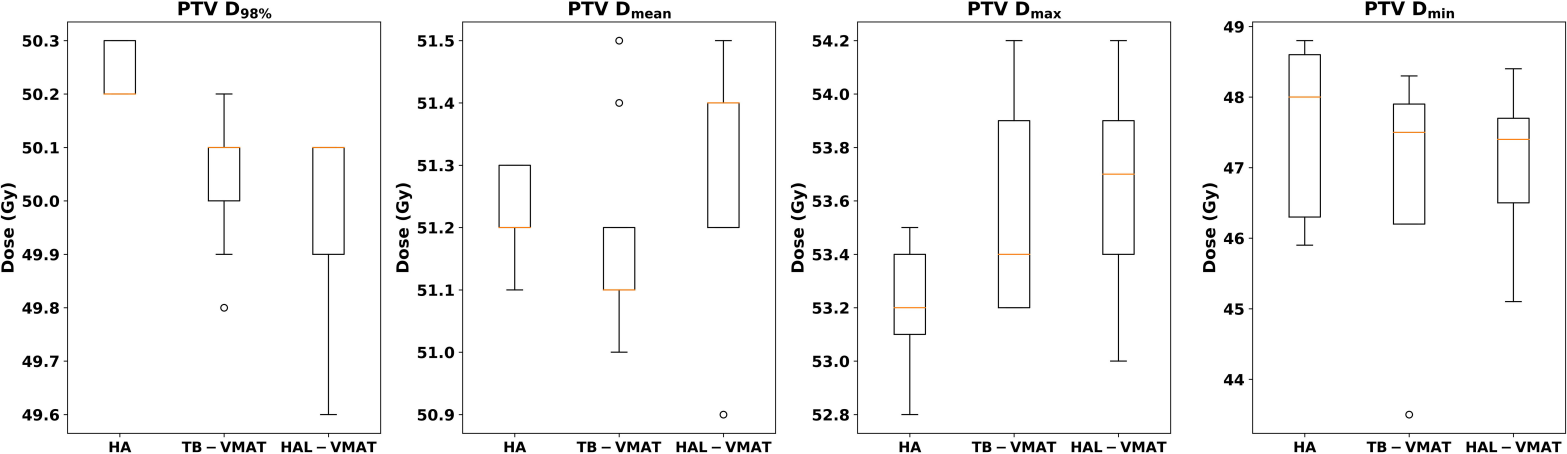
Box plots of dosimetric parameters of D_98%_, D_mean_, D_max_, and D_min_ for PTV of HA, TB-VMAT, and HAL-VMAT plans.

**Figure 3:**
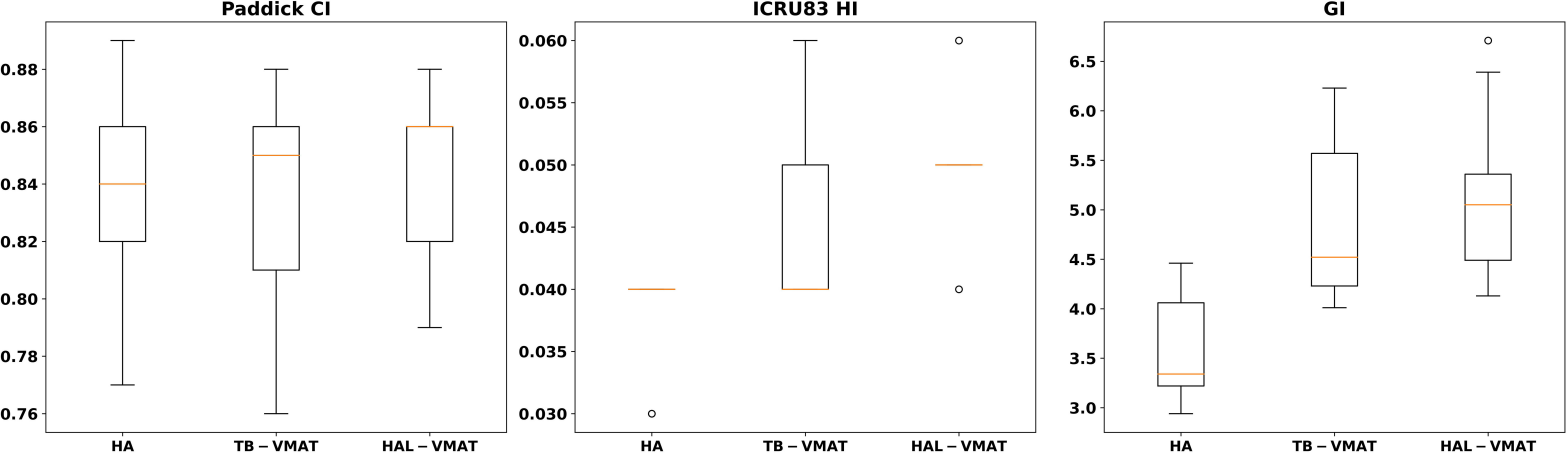
Box plots of dosimetric parameters of PCI, HI, and GI for PTV of HA, TB-VMAT, and HAL-VMAT plans.

**Figure 4:**
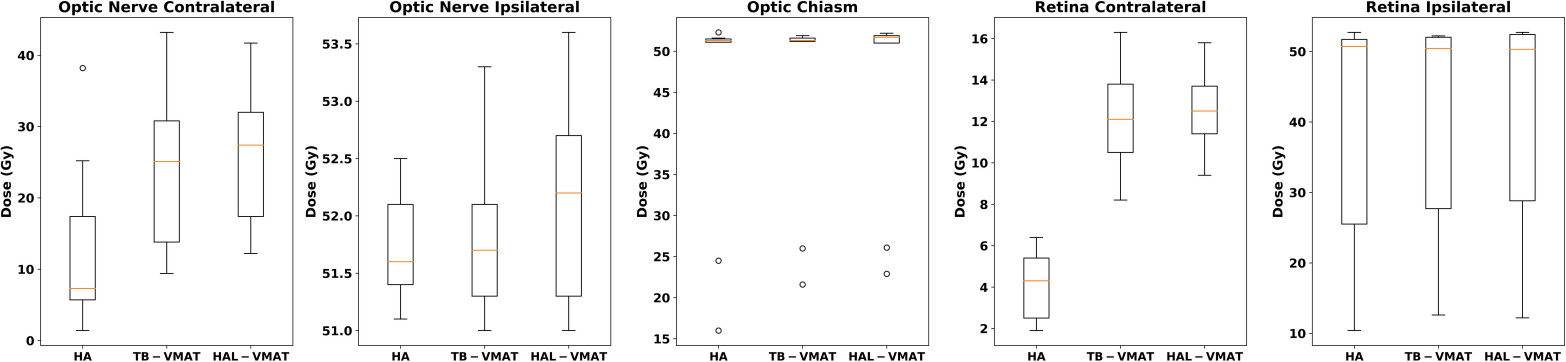
Box plots of the D_max_ of optic nerves, optic chiasm, and retinas of HA, TB-VMAT, and HAL-VMAT plans.

**Figure 5:**
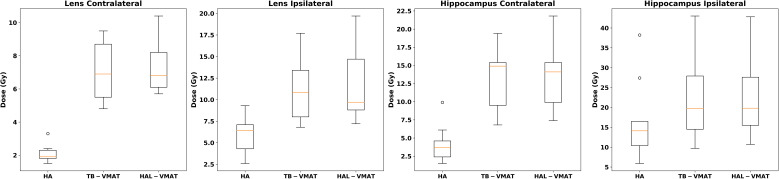
Box plots of the D_max_ of lenses and hippocampi of HA, TB-VMAT, and HAL-VMAT plans.

**Figure 6:**
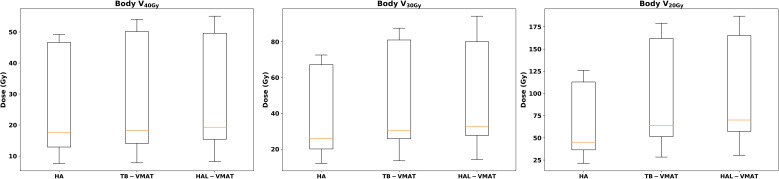
Box plots of the dosimetric parameters of volume received 20 Gy, 30 Gy, and 40 Gy in body of HA, TB-VMAT, and HAL-VMAT plans.

**Figure 7:**
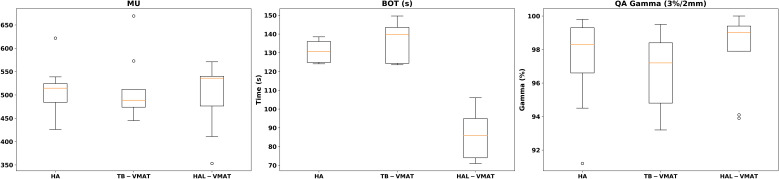
Box plots of MUs, BOT, and QA Gamma of HA, TB-VMAT, and HAL-VMAT plans.

Box plots (**Figures 2**–**7**) were used to illustrate the distribution of key dose metrics across the three treatment plans: HA, TB-VMAT, and HAL-VMAT. Each plot shows the median (orange line), interquartile range (IQR, box), and data range (whiskers), with outliers indicated as individual points. These visualizations provide a clear summary of the data distribution, highlighting central tendency, spread, and inter-group variability among the treatment techniques. Results in **Tables 3**, **4** are expressed as the mean ± standard deviation. Statistical differences among HA, TB-VMAT, and HAL-VMAT plans were assessed using the Wilcoxon signed-rank test, with a significance threshold of p < 0.05 for each pairwise comparison (HA vs. TB-VMAT, HA vs. HAL-VMAT, and TB-VMAT vs. HAL-VMAT).

**Table 3:** Dosimetric parameters for target and OARs.

Structure	Dosimetric parameters	Technique						*p*-value	*p*-value	*p*-value
		HA (a)		TB-VMAT (b)		HAL-VMAT (c)		a vs. b	a vs. c	b vs. c
		Mean	Std	Mean	Std	Mean	Std			
PTV	D_98%_ (Gy)	50.24	0.05	50.04	0.13	49.99	0.17	0.011*	0.004*	0.340
	D_mean_ (Gy)	51.22	0.07	51.18	0.17	51.30	0.18	0.395	0.236	0.092
	D_max_ (Gy)	53.21	0.22	53.57	0.40	53.67	0.36	0.050*	0.017*	0.359
	D_min_ (Gy)	47.63	1.18	46.98	1.50	47.04	1.08	0.074	0.164	1.000
	Paddick CI	0.84	0.04	0.83	0.04	0.84	0.03	0.750	0.572	0.496
	ICRU83 HI	0.04	0.00	0.05	0.01	0.05	0.01	0.020*	0.010*	0.046*
	GI	3.62	0.57	4.84	0.81	5.14	0.90	0.004*	0.004*	0.050*
Lens contralateral	D_max_ (Gy)	2.09	0.56	7.00	1.76	7.41	1.67	0.004*	0.004*	0.055
Lens ipsilateral	D_max_ (Gy)	5.91	2.10	11.29	3.92	11.71	4.42	0.004*	0.004*	0.164
Optic nerve contralateral	D_max_ (Gy)	13.68	12.02	24.22	10.91	25.76	9.72	0.004*	0.004*	0.027*
Optic nerve ipsilateral	D_max_ (Gy)	51.68	0.46	51.81	0.70	52.16	0.89	0.233	0.039*	0.043*
Optic chiasm	D_max_ (Gy)	44.54	13.94	45.34	12.27	45.67	12.03	0.091	0.027*	0.039*
Retina contralateral	D_max_ (Gy)	4.10	1.64	12.14	2.58	12.56	2.21	0.004*	0.004*	0.123
Retina ipsilateral	D_max_ (Gy)	39.49	18.31	41.41	15.47	41.52	15.73	0.164	0.068	0.570
Hippocampus contralateral	D_max_ (Gy)	4.24	2.54	12.92	4.55	13.10	4.69	0.004*	0.004*	0.889
Hippocampus ipsilateral	D_max_ (Gy)	16.73	10.05	22.08	10.88	22.41	10.66	0.004*	0.004*	0.359
Hippocampus bilateral	D_40%_ (Gy)	5.79	2.46	10.19	4.10	9.84	3.67	0.004*	0.004*	0.164
Body	V_40Gy_ (cm^3^)	27.39	17.85	29.50	19.80	30.32	20.03	0.004*	0.004*	0.020*
Body	V_30Gy_ (cm^3^)	40.06	25.54	48.49	31.79	50.88	33.17	0.004*	0.004*	0.020*
Body	V_20Gy_ (cm^3^)	68.72	43.24	97.52	62.39	103.31	64.40	0.004*	0.004*	0.004*

^*^Statistically significant with p value threshold of 0.05.

**Table 4:** Table of MUs, BOT, and QA Gamma of HA, TB-VMAT, and HAL-VMAT plans.

Parameters	Technique						*p*-value	*p*-value	*p*-value
	HA (a)		TB-VMAT (b)		HAL-VMAT (c)		a vs. b	a vs. c	b vs. c
	Mean	Std	Mean	Std	Mean	Std			
Total MUs	510.28	54.19	511.17	70.02	496.42	71.95	0.910	0.426	0.820
BOT (s)	130.97	5.60	135.97	10.36	86.38	13.72	0.054	0.004*	0.004*
QA Gamma (3%/2mm)	97.3%	2.8%	96.6%	2.4%	97.9%	2.3%	0.496	0.426	0.203

^*^ Statistically significant with p value threshold of 0.05.

## Results

**Table 3** presents a detailed comparison of all dosimetric parameters for the PTV and OARs across HA, TB-VMAT, and HAL-VMAT plans. Significant dosimetric differences were observed among the three techniques in terms of target coverage and OAR sparing. For the PTV, HA plans demonstrated a significantly higher D_98%_ than the TB-VMAT and HAL-VMAT plans (p < 0.05), while the D_max_ was significantly lower (p < 0.05). This indicates that the HA plans generated more homogeneous dose distributions compared to the others. For the OARs, the D_max_ values for the HA plans—specifically for both lenses, contralateral optic nerve, contralateral retina, and hippocampi—were the lowest among the three plans (p < 0.05). Additionally, the volumes of the body receiving doses in the range of 20 Gy, 30 Gy, and 40 Gy (V_20Gy_, V_30Gy_, and V_40Gy_) were also significantly lower in the HA plans compared to the other plans.

**Figure 8:**
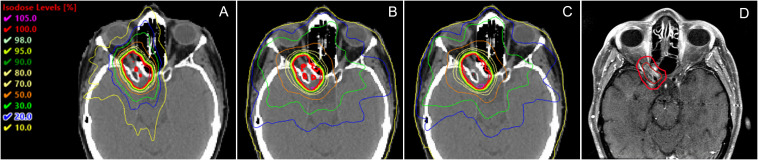
Comparison of dose distributions among **(A)** HA, **(B)** TB-VMAT, and **(C)** HAL-VMAT treatment plans, alongside the corresponding MRI image **(D)** for the same patient. Both the GTV (ONSM) and PTV are outlined in red, illustrating how the lesion circumferentially envelops the right optic nerve.

**Figure 9:**
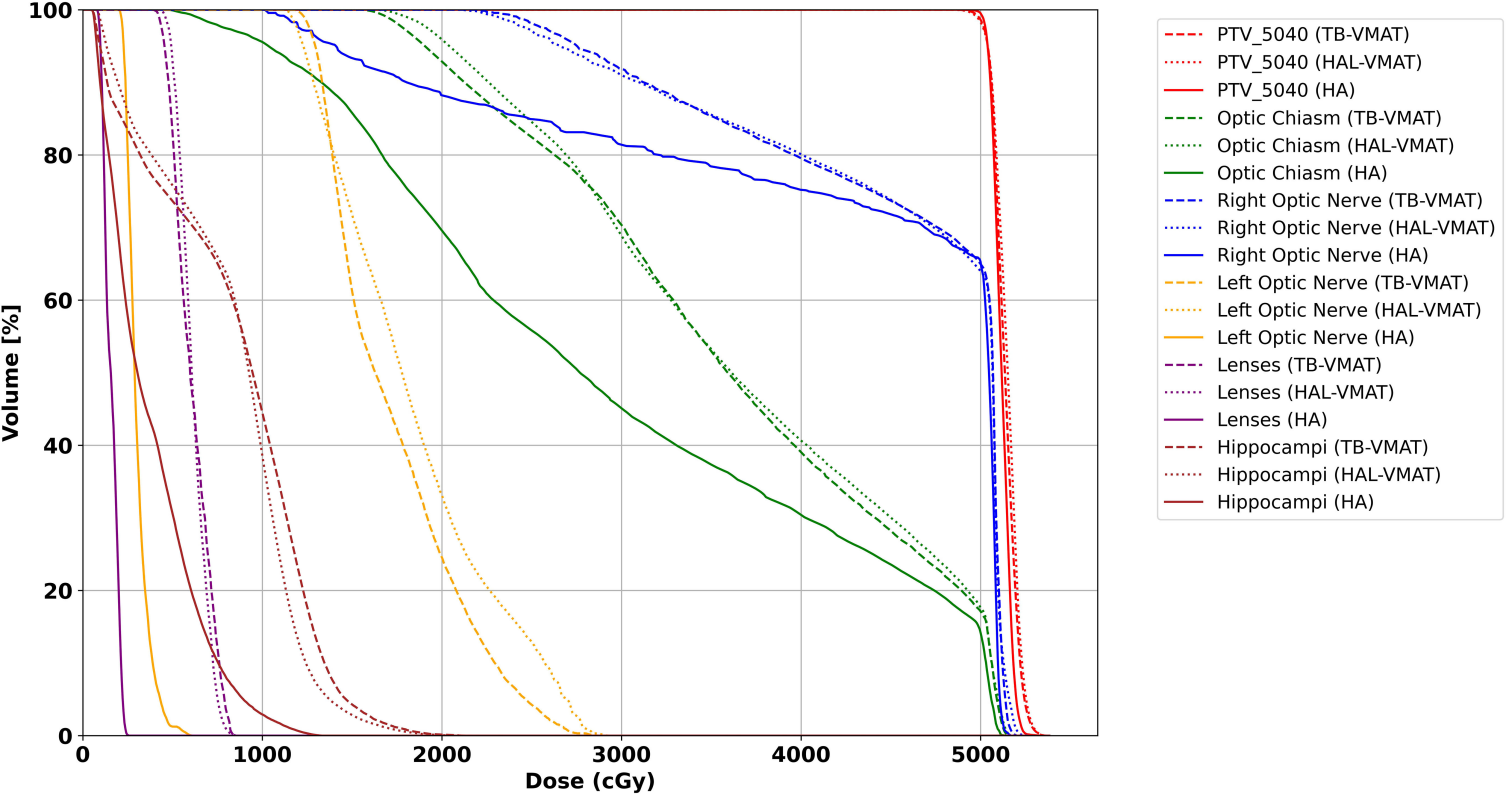
DVH for PTV and OARs for the patient illustrated in **Figure 8**. Solid line = HA, dashed line = TB-VMAT, dotted line = HAL-VMAT.

The isodose distributions among the treatment plans for one patient in axial view are shown in **Figures 8A–C**. The volume of the body receiving 10%, 20%, 30%, and 50% of the prescription dose was lower in the HA plans than in the TB-VMAT and HAL-VMAT plans. As illustrated, the 50% isodose line conformed better around the PTV in the HA plan compared to the others, while the 10% and 20% isodose lines were much more spread out in the TB-VMAT and HAL-VMAT plans. The MRI image of the same patient, with the GTV and PTV contoured in red, is shown in **Figure 8D**. The ONSM can be observed encircling the right optic nerve circumferentially. **Figure 9** shows the DVHs for the PTV and several key OARs—including the PTV, both lenses, both optic nerves, optic chiasm, and bilateral hippocampi—for the same patient. In this case, HA plans ensured comprehensive target coverage and reduced hotspots within the PTV. For OARs located within or immediately adjacent to the PTV (e.g., the ipsilateral optic nerve and optic chiasm), maximum doses were similar across techniques, although HA continued to reduce lower and intermediate dose levels. Conversely, for other OARs (bilateral lenses, hippocampi, and the contralateral optic nerve), HA achieved a significant maximum dose reduction compared to TB-VMAT and HAL-VMAT plans.

**Figure 2** presents the comparison of target coverage parameters: PTV D_98%_ (50.24 ± 0.05 Gy, 50.04 ± 0.13 Gy, and 49.99 ± 0.17 Gy for HA, TB-VMAT, and HAL-VMAT, respectively), PTV D_mean_ (51.22 ± 0.07 Gy, 51.18 ± 0.17 Gy, and 51.30 ± 0.18 Gy for HA, TB-VMAT, and HAL-VMAT, respectively), PTV D_max_ (53.21 ± 0.22 Gy, 53.57 ± 0.40 Gy, and 53.67 ± 0.36 Gy for HA, TB-VMAT, and HAL-VMAT respectively), and PTV D_min_ (47.63 ± 1.18 Gy, 46.98 ± 1.50 Gy, and 47.04 ± 1.08 Gy for HA, TB-VMAT, and HAL-VMAT, respectively). Although the absolute differences of the mean PTV D_98%_ were small among three groups, the HA plans still demonstrated a higher mean PTV D_98%_ with p < 0.05 compared to the TB-VMAT and HAL-VMAT plans. Furthermore, the mean PTV D_max_ for the HA plans was lower compared to the TB-VMAT and HAL-VMAT plans with p < 0.05. The PTV D_min_ of the HA plans was slightly higher than that of the TB-VMAT and HAL-VMAT plans, but without statistical significance (p = 0.074 and p = 0.164, respectively). For PTV D_mean_, the HA plans showed no significant differences compared with the TB-VMAT and HAL-VMAT plans.

In terms of dosimetric parameters related to PCI, HI, and GI, **Figure 3** shows that the HI in the HA plans (0.04 ± 0.00) was significantly lower (p < 0.05) than in the TB-VMAT (0.05 ± 0.01) and HAL-VMAT (0.05 ± 0.01) plans, indicating that the HA plans resulted in a more homogeneous dose distribution within the PTV volume. Additionally, the GI in the HA plans (3.62 ± 0.57) was also significantly lower (p < 0.05) than in the TB-VMAT (4.84 ± 0.81) and HAL-VMAT (5.14 ± 0.90) plans. This means that the HA plans showed significantly faster dose fall-off compared to the TB-VMAT and HAL-VMAT plans, potentially better sparing the adjacent OARs. Meanwhile, the differences in the PCI among the three plans were not significant.

The comparisons of dosimetric parameters for the OARs are shown in **Figures 4**, **5**. Except for the optic chiasm, the ipsilateral optic nerve and the ipsilateral retina, the D_max_ values in the HA plans for other OARs were the lowest among the three plans (p < 0.05). Specifically, the HA plans had lower D_max_ values for the contralateral lens (2.09 ± 0.56 Gy, 7.00 ± 1.76 Gy, and 7.41 ± 1.67 Gy for HA, TB-VMAT, and HAL-VMAT, respectively), ipsilateral lens (5.91 ± 2.10 Gy, 11.29 ± 3.92 Gy, and 11.71 ± 4.42 Gy, for HA, TB-VMAT, and HAL-VMAT, respectively), contralateral optic nerve (13.68 ± 12.02 Gy, 24.22 ± 10.91 Gy, and 25.76 ± 9.72 Gy, for HA, TB-VMAT, and HAL-VMAT, respectively), contralateral retina (4.10 ± 1.64 Gy, 12.14 ± 2.58 Gy, and 12.56 ± 2.21 Gy, for HA, TB-VMAT, and HAL-VMAT, respectively), contralateral hippocampus (4.24 ± 2.54 Gy, 12.92 ± 4.55 Gy, and 13.10 ± 4.69 Gy, for HA, TB-VMAT, and HAL-VMAT, respectively), and ipsilateral hippocampus (16.73 ± 10.05 Gy, 22.08 ± 10.88 Gy, and 22.41 ± 10.66 Gy, for HA, TB-VMAT, and HAL-VMAT, respectively). **Figure 6** shows that the volumes of the body receiving doses of 20 Gy, 30 Gy, and 40 Gy (V_20Gy_, V_30Gy_, and V_40Gy_) were also significantly lower (p < 0.05) in the HA plans compared to the TB-VMAT and HAL-VMAT plans.

**Table 4** and **Figure 7** present a comparison of total MUs, BOT, and QA gamma passing rates for the HA, TB-VMAT, and HAL-VMAT plans. There were no statistically significant differences in the total MUs and QA gamma passing rates among the three techniques. Regarding BOT, the HA (130.97 ± 5.60 s) and TB-VMAT (135.97 ± 10.36 s) plans required significantly longer (p < 0.05) beam-on times than the HAL-VMAT plans (86.38 ± 13.72 s). However, considering the time required for patient setup and cone-beam computed tomography (CBCT) imaging and registration, the overall treatment time would be typically longer than the BOT difference.

## Discussion

Only a few studies have explored the use of advanced VMAT techniques for ONSMs with conventional fractionation and SRT approaches (3, 6, 12). ONSMs usually surround or involve various critical OARs, such as the optic nerves, optic chiasm, and retinas; thus, sophisticated radiotherapy techniques are required to maximize treatment efficacy while minimizing the risk of radiation-induced side effects. In this study, we conducted a comprehensive comparison of the dosimetric quality of HA and two other advanced techniques for radiotherapy of ONSMs. To our knowledge, this is the first study to evaluate the dosimetric performance of HA planning with that of TB-VMAT and HAL-VMAT planning—three advanced representative techniques—in radiotherapy for ONSMs. All retrospective plans in this study were clinically acceptable and demonstrated dosimetric outcomes equal to or better than the originally delivered plans. The results showed that all three techniques provided sufficient target coverage, but HA performed better in dosimetric parameters PTV D_98%_, HI, and GI. HA also minimized the radiation dose to OARs more effectively and reduced dose spread (lower V_20Gy_, V_30Gy_, and V_40Gy_ for the entire body) compared to TB-VMAT and HAL-VMAT.

The components of the visual system—the optic nerve, optic chiasm, and retina—are serial organs with known radiation dose tolerance thresholds (24). Becker et al. (25) reported that in the absence of prior damage from tumors or surgery to the optical pathways, and when single radiation doses of less than 2 Gy and total doses of 45–50 Gy are used, the risk of optic neuropathy is below 2%. However, the risk of optic neuropathy rises to 5% when the total dose increased to 54 Gy (26, 27). Furthermore, although radiotherapy for ONSMs generally provides good tumor control, tumors may recur in approximately 5% of cases (3, 28, 29), usually occurring in patients with larger target volumes. Bilateral tumors occur in about 5–10% of ONSM patients, caused by overgrowth from the opposite side (3). Thus, it is important to keep the radiation dose to the visual system as low as possible without compromising target coverage. In this context, HA can be an effective treatment technique in the regimen of regular fractionation VMAT/IMRT, as demonstrated by our study that HA resulted in the lowest doses to the visual system: the lower D_max_ values for the contralateral optic nerve (43.5% reduction compared to TB-VMAT and 46.9% reduction compared to HAL-VMAT), bilateral lenses, and contralateral retina, as well as the significantly lower GI (**Table 3**).

Based on pivotal dose-effect meta-analyses (30, 31), clinical threshold doses for vision-impairing cataracts requiring surgery were identified as 10 Gy and 18 Gy, resulting in 5% and 50% incidence rates at 5 years post-irradiation, respectively. Nguyen et al. (32) reported that through lens dose-response modeling in treating patients with retinoblastoma, they established a mean lens dose threshold of 7 Gy to keep cataract risk below 25%. In radiotherapy, the lens dose is usually minimized according to the “as low as reasonably achievable” (ALARA) principle. In our study, we demonstrated that HA plans outperformed TB-VMAT and HAL-VMAT plans in terms of lens dose reduction. Specifically, compared to TB-VMAT plans, HA achieved an average 47.6% reduction in ipsilateral lens D_max_ dose (from 11.29 Gy to 5.91 Gy) and a 70.2% reduction in contralateral lens D_max_ dose (from 7.00 Gy to 2.09 Gy). Similarly, compared to HAL-VMAT plans, HA achieved an average 49.5% reduction in ipsilateral lens D_max_ dose (from 11.71 Gy to 5.91 Gy) and a 71.8% reduction in contralateral lens D_max_ dose (from 7.41 Gy to 2.09 Gy). These results indicated that HA can significantly reduce the risk of radiation-induced cataracts in patients undergoing radiotherapy for ONSMs, especially for the ipsilateral lens.

Several studies have reported that declines in neurocognitive function—specifically deficits in learning, memory recall, and spatial processing—are associated with radiation-induced damage to the hippocampi (33–35). Gondi et al. investigated the association between hippocampal dose and long-term neurocognitive function and discovered that a dose to 40% of the bilateral hippocampi (D_40%_) greater than 7.3 Gy predicts the risk of long-term impairment in list-learning delayed recall after radiotherapy (36). In our study, the mean D_40%_ of the bilateral hippocampi in the HA plans was 5.79 Gy, which is below the 7.3 Gy threshold reported by Gondi et al. In contrast, the TB-VMAT and HAL-VMAT plans had D40% values that were 75.9% and 69.9% higher, respectively, thus exceeding this threshold. Thus, HA plans have the potential to preserve neurocognitive function in patients with ONSMs undergoing radiotherapy.

In this study, the HA plans used conventional fractionation with a total of 28 fractions. Recently, there has been an increase in studies focusing on SRT for ONSMs (3, 6, 10). In 2021, Senger et al. (3) reviewed the results of five studies on 84 patients with ONSMs treated with SRT or SRS via CyberKnife or Gammaknife. The total treatment doses ranged between 10 and 25 Gy in 1–5 fractions, with overall local tumor control rates between 93% and 100%. In their own cohort of 25 patients with 27 ONSM lesions, they reported a local tumor control rate of 96% with 20–25 Gy delivered in 4–5 fractions, and stable or improved visual acuity in 100% of patients (90.0% stable and 10.0% improved). Vakharia et al. (37) reported a case of using salvage single-session SRS with a dose of 15 Gy for a recurrent ONSM patient. HA utilizes a frameless masking system (Encompass) (38) with an optional add-on of surface-guided imaging system (39, 40) for patient immobilization and tracking during setup and treatment, which is crucial for patients undergoing SRT or SRS. A 2020 study by Komiyama et al. (41) demonstrated that Encompass provided patient immobilization with adequate accuracy during HA treatment, with absolute maximum intra-fractional motion values of less than 1 mm along the superior-inferior and left-right axes and less than 1° along the yaw axis. Ohira et al. (42) reported that the margin compensation for intrafractional motion error was less than 1 mm for Encompass. Covington et al. (43, 44) reported that using AlignRT^®^ (VisionRT, London, UK) or IDENTIFY™ (Varian Medical Systems) surface-guided imaging systems during frameless intracranial SRS with HA offers sub-millimeter accuracy with real-time measurements and no loss of treatment efficiency. Thus, with the Encompass immobilization and surface-guided imaging systems, treating ONSMs with HA in an SRT or SRS regimen is feasible and provides greater delivery confidence in high-dose per fraction. Additionally, the use of a surface-guided imaging system in HA treatment for ONSMs helps streamline the setup process by reducing the time required to achieve accurate positioning and minimizing reliance on CBCT scans for setup verification. This reduces the need for a second CBCT scan if there is significant angular mismatch, thereby decreasing the overall radiation dose to the patient and enhancing treatment efficiency. In our study, a 5-7mm margin from GTV to PTV expansion was used for the three groups. The margin could be further reduced when using HA TrueBeam machine equipped with PerfectPitch 6 degree-of-freedom (DoF) couch, Encompass immobilization system, and surface-guided imaging system. Using a smaller margin, the total irradiated volume will be decreased, and the adjacent OAR doses will likely be reduced. With the growing trend of managing ONSMs using SRT or other low-fraction regimens, HA in this context not only remains feasible but also provides enhanced confidence for high-dose-per-fraction treatments.

The mean BOT in the HA plans was similar to that of the TB-VMAT plans, and both were about 1.5 times longer than that in the HAL-VMAT plans. This is because the Halcyon machine has an increased gantry rotation speed compared to TrueBeam machine, and the dose rate of the HAL-VMAT plans is 800 MU/min, which is higher than the dose rate of 600 MU/min for the HA and TB-VMAT plans. In addition, the total time of the automated couch rotation was around 1 minute for the HA plans. However, in terms of overall treatment time, the automated delivery of HA’s noncoplanar beam arrangements and the virtual dry run for collision checks—which possibly eliminates the need for physical dry runs prior to treatment—provide a significant decrease in total treatment time. Thus, the overall treatment time for HA plans can still be accommodated within the same treatment time slot as the other two coplanar VMAT techniques. Consequently, utilizing HA with automated non-coplanar planning will require fewer clinical resources overall, potentially increasing efficiency and reducing staff workload.

There are some limitations in this study. First, ONSM is a rare benign tumor, and only a subset of patients receive radiotherapy. Previous reports have also included relatively small numbers of patients undergoing radiotherapy for the same reason. Thus, there are only nine patients in our study, and the small sample size must be considered when analyzing the results. Moreover, given the small patient cohort and the low incidence of radiation-induced adverse effects, no adverse radiation events were observed in the clinically treated cases. Second, the HA, TB-VMAT, and HAL-VMAT plans were generated using the same optimization parameters in this study to ensure a fair comparison. However, in practice, the optimal optimization parameters may differ based on the technique used and specific clinical factors such as tumor location and size. Third, we used a TrueBeam machine with a Millennium MLC for HA planning instead of a High Definition MLC (HDMLC), which could potentially improve target coverage and conformity, due to the smaller MLC size on the HDMLC machines. However, since it is generally observed that more facilities are equipped with machines using Millennium MLCs than HDMLCs, and as noted by Bossart et al. (45), HDMLC should be considered for treating lesions smaller than 1 cc with HA, given that the majority of ONSM targets exceed this size, our study remains highly applicable. Lastly, this study compares HA, a noncoplanar VMAT technique, with two coplanar VMAT techniques, which may introduce some bias. Noncoplanar VMAT plans can typically outperform coplanar VMAT plans in terms of target coverage and OAR sparing. However, given that little information is available about noncoplanar VMAT techniques for ONSMs in recent studies (46, 47), the comparison remains valuable. Moreover, HA enables automatic treatment delivery with couch rotation, saving time and making the total treatment time comparable to that of coplanar plans. Despite these limitations, our quantitative data provide meaningful information for ensuring comprehensive target coverage and reducing hotspots within the target while better protecting OARs in radiotherapy for ONSM patients. These advantages suggest that the HA technique should be considered for radiotherapy treatment of ONSMs and should be clinically evaluated more in the future.

## Conclusion

Our dosimetric results demonstrated that HA plans provided superior target coverage (higher PTV D98%), improved dose homogeneity (lower HI), sharper dose gradients (lower GI), and reduced hotspots within the PTV compared to TB-VMAT and HAL-VMAT plans. Furthermore, HA plans significantly reduced doses to the contralateral visual system as well as the hippocampi. Additionally, its template-based planning, streamlined setup, and automated delivery, along with the use of Encompass and surface-guided imaging system to improve delivery accuracy, make HA an effective approach for radiotherapy in patients with ONSMs. These advantages suggest that HA should be considered for treating ONSMs with conventional fractionation in addition to its use in SRS/SRT.

## Data Availability

The raw data supporting the conclusions of this article will be made available by the authors, without undue reservation.
